# Macon Medical Society

**Published:** 1884-11

**Authors:** 


### Society Reports.

### MACON MEDICAL SOCIETY.

Reported for THE ATLANTA MEDICAL AND SURGICAL JOURNAL.

STATED MEETING, September 16, 1884.

Dr. C H. Hall, president, in the chair.

Dr. Howard Williams opened the discussion by reading the following paper:

### THE IMPORTANCE OF FREQUENT POST MORTEM EXAMINATIONS.

In the investigation of any important subject, in order to gain thorough and comprehensive knowledge, every avenue of inquiry must be well explored. These investigations must be personal, or they have not that personal value which makes them of practical use. The light received from the writings of others is reflected, and is of value alone in giving the observer a superficial knowledge of what he may discover, if he takes the torch in his hand and looks into every recess.

In our profession this is pre-eminently true. To gain practical knowledge of disease, the physician must first in the text-books learn what he is to observe, and then himself prove the truth of what he reads by untiring industry and personal observation. The knowledge gained from the careful investigation of a single case is of infinitely more practical value than that from studying the writings of a hundred authors. Facts must be not only collected from every source, but must be sifted, weighed, and their inter-dependence determined, before their individual value can be assigned.

To recognize the manifestations of disease, every sense we have must be employed, and the aids to these senses which art has devised should be called into play. Sight, hearing, smell and touch should give their information; instruments of precision, chemical reactions, microscopical investigations, and post-mortem observations should combine to throw light on the morbid process called disease. Every fresh acquirement tends to enlarge our powers of insight, and we fail to improve our knowledge if we neglect these. As we ascend the mountain, our field of vision broadens, and we are better able to judge of the country which lies at our feet; so in our profession – the more numerous our aids to investigation, the better able are we to reason upon the morbid processes at work.

I have of late been impressed with the need of post-mortem examinations in our city. Accustomed to frequent examinations of the dead during my residence in the hospital at Philadelphia, I have missed this privilege since locating in Macon. I have frequently seen this necessity, and have wished that the local opposition to its importance might be broken through, and the desire for improvement in our knowledge of disease receive fresh impetus from this means of investigation.

I have conducted only two post-mortem examinations since my return, and these were obtained after much opposition. The obscurity of the cause of death in both instances warranted the examinations. In the first, a difference of opinion in consultation between intussusception and hemorrhage into the peritoneum, led to the post-mortem, which disclosed that the latter had taken place from the rupture of a tumor of the kidney, producing death. The other, a case of of obscure heart disease, which the post-mortem disclosed to be universal pericardial adhesions.

We are all earnestly searching after higher medical education and greater exactness in the history, nature and treatment of disease, in order that we may better meet the practical demands of our profession. We use every means in our power to arrive at correct diagnosis, prognosis and treatment, why not go farther and send our inquiries into the pathology and morbid anatomy of the diseases which we meet? By seeing the results of disease in the various organs and tissues, we are enabled to draw better conclusions of what the disease is and the demands in treatment, and when we meet similar cases we have a better idea of what are the conditions present, and the best means to combat the existing trouble. How can one conceive the appearance of an organ, swollen with the products of inflammation, who has never seen the actual condition itself? Words but faintly describe it; photographs and painted plates give only indistinct and insufficient ideas of what is present. But when the condition is seen in the organ itself, the reason why such and such symptoms and signs are present, and the dangers to which sufferers from the disease are exposed, are apparent, and the mind goes out in inquiry for means to relieve the existing condition and how best to meet the dangers attending such crippling of an organ. The physician is no longer a mere prescriber, but becomes the earnest student of disease and the practical worker for the good of his patients. Fresh impetus is given to self-education, and he rises in the field of knowledge.

It is said we learn most by recognizing our mistakes. After every moral and mental depression, true manhood reacts with renewed energy and greater earnestness to overcome obstacles, and at length gains success. Disease of one organ is supposed to be present, the patient dies, and at the post-mortem the disease is found to be in an entirely different organ. Shocked at the mistake, the history of the case is reviewed; obscure symptoms and signs present during life are now seen to have plainly pointed to the diseased organ; to avoid error again, the disease is restudied. The practitioner, instead of drawing hasty conclusions, receives an impetus to make careful examinations, and reasons upon his cases, and thus rises in the world of medical thinkers.

These and many others are the personal reasons for post-mortem examinations. To raise the standard of medical observation and knowledge in the South is a reason for more frequent post mortems in our midst. I do not intend to place a low estimate upon the acquirements of the medical profession in the South, nor could I with truth say it is low. With the advantages our profession enjoys, it has kept up wonderfully in medical advancement, and compares favorably with that of other sections, yet we must admit it has not taken the position it should hold. There are earnest thinkers in the South, who, if they had the advantages, would hold high rank in the medical world. How can post-mortems aid them? Diseases in general are the same everwhere, yet they are variously affected, as by civilization, occupation, hereditary tendency, race, climate, etc. New features in diseases are constantly presenting themselves, and are constantly being brought to light by investigations elsewhere; why should not the southern practitioner become an active investigator in this direction? With the growth of civilization and the requirements of a progressive world, the demands upon the nervous system are more imperative, and an increase in the diseases of the nervous system is a natural result. The luxuries of mankind and the indulgences of the pleasures of life have grown with the growth of civilization, leaving their marks on the constitution of man, and changing the type of nervous diseases.

Occupation has its influence on disease. New industries are constantly springing up and affect not only the mental character of our people, but their constitutional tendencies. The generation of electricity, fumes of poisons employed in the dyeing process, vapor of steam, fumes from the manufacture of gas, dust from the cotton loom, all are taken into the system and no doubt exert a deleterious effect upon the organs of the body and modify their diseases.

Hereditary tendency modifies diseases, and the growth of civilization and new and varied occupations must set up new tendencies. The question of the effect of race on disease is one of importance and one that should be of peculiar interest to us. The changes wrought by the war in the negro have made him a fruitful source of study. The changes in his occupations, social conditions, hygienic surroundings, illicit relations with the white race, must certainly affect the type of diseases peculiar to the race and develop new tendencies. What a field of study and observation to pursue, and much practical knowledge can be brought to light by investigating the effect of their liberty on their diseases and diseased organs.

The effect of climate on disease is a question of importance. The diseases of the South require, I feel, a better classification, and their types should be more clearly defined. The southern fevers open a wide field of investigation and are fruitful to an earnest observer. The changes in the organs, found by post-mortem examinations, would no doubt throw much light upon this important subject. Perhaps there exist among the continued fevers we are meeting, the identity of which we are in doubt, several fevers peculiar to our section, which have heretofore remained undescribed. The identity of the continued fever, which for want of a better name we designate "so-called typhoid," may be decided by post mortem examination. If it be true typhoid or enteric, the pathological changes will reveal it, or if not, its true character will be determined.

Malarial poisoning modifies our diseases and changes are made by it in the organs of the body. The malarial element is present to a large extent in the South, and clearer ideas of its character and influence should be sought by us. Our climate affects the diseases of the liver, spleen, bowels and other organs; and some of the diseases of these organs are found in their clearest type in the South.

Questions of these and similar character are constantly coming up for discussion. Now if post-mortem examinations will throw light upon these questions, is it not our duty, if we wish to be progressive, to resort to them more frequently? They add to our knowledge of disease and enable us to appreciate pathological subjects discussed in advanced medical journals, for we are thrown into a more intimate understanding with our leading medical thinkers. They enable us to discriminate between truth and theory, and appreciate why that which is observed in one section of country will not be tenable in any other. Our medical knowledge to be practical must be based upon observations made in our midst, and we should not be too ready to receive everything as truth for us until it is demonstrated under the existing conditions by which we are surrounded. If we enjoyed more frequent post mortem examinations in the South, our medical schools and teachers would be better prepared to instruct students in the nature, character, and process of disease. Add to clinical teaching, which is admittedly the highest form of medical education, pathological examinations, and our medical student will be better prepared to enter into the active practice of his profession. In the one he is thrown into intimate relation with his patients, on the other he is as intimate with disease. Give the southern student all the advantages possible of studying southern diseases and he is better prepared to treat disease as he finds it in the South.

Improvement in our mortuary statistics is a very serious reason for frequent post-mortem examinations. But little use was made of vital statistics until the present century and much of the progress in medicine during this century, is due to the direct use of these statistics in pathology, etiology, and therapeutics. Mortuary statistics are employed to determine the limits of mortality in a community; the death rate of a community without reference to sex, age, or disease, expresses its health and its sanitary condition. Investigation of such statistics discloses the age, sex, and class which gives the highest death-rate, the district of the community in which most deaths occur, the season of the year during which the largest number of deaths occur, the character and class of diseases causing the majority of deaths, and the causes producing a too-high death-rate of a particular period. The value of these statistics depends upon the uniformity of facts observed and the accuracy with which observations are made.

The medical profession bears a grave responsibility for the accuracy of such statistics, for the records may greatly affect the actions of a community, taking records in faith in this accuracy of observation, in measures to protect the public health. Carelessness of diagnosis will give error in these records, and indifference in ascertaining the true causes producing death may seriously affect sanitary legislation. The physician, in filling out his certificate, should not consider it a mere matter of indifference and a useless piece of red tape to be hastily gone through and forgotten. He should regard it, as it truly is, an effort to arrive at definite knowledge of the dangers surrounding the health of the community, so that by wise sanitary measures the dangers may be removed. He should be careful, therefore, to be as accurate as his opportunities permit, in arriving at the true cause of death; it is not sufficient to record death from some prominent symptom common to several diseases, as is so frequently done. In doubtful cases he should insist on the necessity of post-mortem examinations, urging as a plea, the protection of the public health and the health of the individuals of the family in which the deaths occur. Sanitary legislation and efforts to remove causes supposed to affect either the present or future health of a community, may and certainly do not meet the approbation of all men, yet their disapproval is no reason why they should flippantly say "it is all stuff and nonsense," nor is it sufficient ground for indifference on their part to professional duties to the public.

But the medical profession is not wholly responsible for all inaccuracies in vital statistics. The sanitary laws themselves are often at fault. Often deaths occur in a community where, because of extreme poverty, ignorance, or indifference on the part of friends, no physician had been in attendance. This state of things is frequently the case in our own community, owing to the poverty of the lower class and the ignorance and indifference of the negro. A wise sanitary law would provide that all such deaths occurring in a community should be followed by post-mortem examinations in order that statistics may be accurate.

Again, sudden deaths are constantly occurring in a community; a coroner (who usually is ignorant on all medical subjects) is called; he impanels a jury, and they, after taking the facts in the case, render a verdict of death from cause unknown, death from natural causes, or death from heart disease, and that ends it. It would be wise, instead of this state of affairs, to have as coroner, or coroner's physician, an intelligent medical man, capable of conducting such examinations, and provide that all coroners' inquests be attended by post-mortem examinations. In this way again our mortuary statistics would improve in accuracy and become of value.

If we examine the mortuary statistics of our city we will see the grossest inaccuracies. Errors in assigning the causes of death are frequent, sometimes showing the most unpardonable carelessness of observations. But I am inclined to think that the members of our profession, when they recognize the necessity of correct mortuary statistics, will be careful to avoid errors of this character. I wish to call attention for a moment to the mortuary reports of our city in order to show of what little value they can possibly be, owing to the large number of deaths from unknown causes registered each year. The records of 1882 show two hundred and forty recorded deaths in the city; of this number the causes of death in thirty-eight, or nearly one-fifth were unknown. In 1883, there were three hundred and twenty-seven deaths recorded, of which ninety-three are registered, cause of death unknown, or nearly one fourth of the entire death record for that year. Up to the 31st of July of the present year, one hundred and forty-two deaths are recorded, of which fifty-one died of unknown causes, or one-third of the deaths registered. Of what practical use can such records be? No correct deductions can be drawn from them. They do not furnish a reliable basis upon which to introduce sanitary reform and measures protecting the public health. To make them accurate and enhance their value, all deaths in which the nature of the disease causing death is doubtful, should be subjected to a post mortem examination. All cases of death in which no physician has been in attendance, and all sudden deaths, should be subjected to the most rigid examination by a competent physician appointed for the purpose. By these means our statistics will be improved, good sanitary measures to eradicate as far as possible the causes of death in our community put in force, and in consequence of this, an improvement in the health of the city.

Dr. J. Emmett Blackshear said:

MR. PRESIDENT—I regard the essay just read by Dr. Williams as timely, and rise simply to thank him for it. The importance of post-mortem examinations cannot be over-estimated. Mortuary reports, to be of value, must, as the Doctor says, be accurate. When physicians fully understand the importance of correct diagnosis, and learn to avail themselves of every means in their power to arrive at such diagnosis, mere symptoms of disease will no longer be given as causes of death. I had intended to mention this matter in my next annual report as chairman of the Board of Health, and also to suggest that provision be made for post-mortem examinations in all cases of death reported from causes unknown, and shall take pleasure in quoting the views of Dr. Williams in that report.

Dr. K. P. Moore said: I take occasion to express my hearty indorsement of Dr. Williams' paper. It is well written, and strikes at an important subject. I believe that the reason that so few post-mortem examinations are made, is more the fault of the doctors than the laity. The people need educating on this question; and if a proper and judicious effort was made by the doctors, consent could much more frequently be obtained than is supposed, to a post-mortem. The older we get in the profession the more indifferent we grow upon this subject. We become busily engaged with the duties of an active professional life, and feel that we hardly have the time to spare to make these examinations. Our youthful ardor and devotion to scientific explorations cool off as the tiresome cases and labors of active life come over us; and we are disposed to conclude that the opposition on the part of family and friends would be so great that it would not be worth our while asking for a post-mortem, when in fact the family and friends would often be only too glad to be able to say with certainty what was the cause of death. I think the subject one of no little importance, and congratulate Dr. Williams upon a move in the right direction, and commend the idea with emphasis to the serious consideration of the Society.

Dr. C. H. Hall said: I recognize the frequent need of post-mortem examinations, and believe that were we to urge the importance of post-mortem examinations in doubtful cases, the friends of the deceased would willingly yield to the request. It is surprising how often the family of a deceased patient show extreme anxiety to know the true cause of death. A physician, however, who is engaged in a large active practice will not have time for post-mortem examinations. If some one not so busily engaged would take hold of the matter no doubt opposition would finally pass away.

Dr. H. McHatton said: I have heard the paper of Dr. Williams with great interest and look upon it as one of the most important papers read before this Society since I have been a member. It seems to me that we cannot keep up with the times in our profession without post-mortems. Looking over the mortuary reports of 1882 and 1883 we find five hundred and sixty-seven deaths; one hundred and thirty-one from causes unknown, leaving four hundred and fifty-seven. Of these, twenty-one died of different symptoms: Dropsy 13, ascites 1, jaundice 3, uræmia 4 Of the remaining four hundred and sixteen, 62 died of various congestions, 1 of disease of head and 1 of complicated. Among the congestions we find 29 congestions of the brain. Gentlemen, our lack of post-mortems makes us careless in our diagnoses. We all know that dropsy and uremia are symptoms, and that idiopathic congestion of the brain as a disease to itself is rare.

I have been informed that one of our practitioners has had three deaths from dislocation of the spleen into the right iliac fossa. If he had had a post-mortem on the first he probably would not have had the last two.

We have had five coroner's juries in the city lately. Three of their cases died of natural causes, but cause unknown; one died of heart disease; one (a girl who had never menstruated) of natural causes—suppression of menses.

I enter a most earnest plea for post-mortems. When any of us have a case that is at all uncertain, let us do all in our power to get as much light on it as a post-mortem will give, and we certainly will be more than paid for the work by our increased knowledge.

After discussing the above paper, the regular subject for the evening, syphilis, was taken up. Dr. J. P. Stevens said: I consider it of the first importance in the treatment of syphilis to discriminate between the characteristics of the chancre and chancroid. The latter, when left to itself, is a self-limiting disease, and finally will result in a cure without mercury, under judicious hygienic conditions. The ulcer is auto-inoculating in the properties of its virus, and usually not co-existent with the presence of inguinal buboes. The vehicle of the chancroidal virus is the secretion of the ulcer and simple abrasion of the cutaneous or other surface is sufficient to generate a chancroidal ulcer by contact. Chancroid being entirely local in its operation upon the system, and not followed by any secondary or tertiary symptoms, it is all-important, in order to make a satisfactory prognosis, that our diagnosis should be certain. The diagnostic peculiarities of chancre and chancroid should therefore be closely and carefully studied. The chancroid is always derived from a chancroid. It is generally multiple by diffusion to the circumjacent tissue. It presents the aspect of an excavated, sharply cut ulcer perforating the thickness of skin or mucous membrane, and its edges well defined, as if incised with a punch, soft, adherent to the subjacent tissue and not movable, as is the chancre. Its surface is flat, with a copious purulent secretion without the hardened base of chancre. It is slow in healing, continuing for weeks and months, unless it is broken down by vigorous cauterization in the incipiency of its formation. Chancre is always derived from a syphilitic lesion. It is not auto-inoculable by the diffusion of its secretion; usually single, sometimes multiple from its incipiency. It presents a hardened base, movable upon the subjacent tissue. Its surface is usually smooth, sometimes its edges are sloping and hard and its secretion is scanty. All the inguinal glands on one or both sides become enlarged and indurated, and are very difficult to be discussed. Chancre is a constitutional affection and one attack for the most part secures an immunity from a second attack. Unless the most vigorous and appropriate tr atment is pursued, secondary symptoms almost invariably follow in about six weeks. Chancroid at its commencement where it is observed outside of the urethra, vagina, or rectum, upon the prepuce or glans, can be broken down by severe cauterization. Its morbid tissue should be destroyed by cauterization with nitric acid or chloride of zinc. Nitrate of silver is too superficial in its effects and cannot be relied on to accomplish a cicatrization of the ulcer.

In primary syphilis mercury should be employed and pushed until the system becomes thoroughly under its influence, but we should avoid salivation, as this process tends to breaking down of the blood, diminishing its red as well as white corpuscles, and thus interfering with the proper and wholesome alimentation of the tissues. In secondary syphilis the bichloride of mercury in minute doses, 1-30 to 1-40 of a grain with elix. calasaya exerts a powerfully recuperative agency, greatly augmenting the number of the oxygen-bearing corpuscles, and improving the digestive functions, thereby promoting the nutrition of the tissues and thus increasing the weight of the body. This process of treatment should be continued for months, and often for years. Compounds of iodine and potash seem to exhibit marked curative properties in the resolution of gummata, nodes, and mucous patches. But the iodide of potassium should be pushed in large doses until the system appears to be saturated with the salt.

Dr. K. P. Moore: "Dr. Stevens, did you state that you always cauterize chancroids?"

Dr. Stevens: "Yes, sir; I think it the quickest way to destroy them. Sometimes chancroids become exceedingly virulent, causing great destruction of tissue, and lasting for months. If, however, they are cauterized early, they soon heal over and are not followed by bubo, for the cautery destroys the virus."

Dr. W. C. Gibson: "What strength do you use the chloride of zinc?"

Dr. Stevens: "I use chloride of zinc and flour, equal parts, made into a paste by the addition of alcohol, drop by drop until the proper consistence is secured."

Dr. Moore: "I agree with Dr. Stevens, and would emphasize the importance of care in the outset in making a correct diagnosis of syphilis. To illustrate the importance of a correct diagnosis in the outset, I will report a case of a young man in high life who applied to me with what was diagnosed a simple chancroid. The young man reported that three or four days previous to his appearance, he had been liable to venery, by visiting a house of ill-fame. The shortness of the period of incubation, together with the mild, soft, blister-like character of the abrasion, and the entire absence of the clean-cut edges and hard base usually seen in chancres, led to the diagnosis of chancroid. The development of two other similar abrasions within the next four or five days in the same region, carrying with it the auto-inoculable doctrine of chancroid, confirmed the diagnosis; and the patient was confidently assured of the fact that his trouble was purely local, and that he need apprehend no fears of constitutional contamination. The local trouble was mildly treated and yielded kindly to treatment. In due course of time, secondary manifestations cropped out in all their bloom and beauty. Two or three other cases of a similar character, but with some less clean-cut symptoms, have occurred in my practice within the last five or six years. As to the treatment and general management of a case of syphilis, I have nothing new to offer. I follow the plan laid down by Van Buren and Keys more closely than any other."

Dr. C. H. Hall: "Do you think anything would have been gained by being able to know from the outset the true character of the case?"

Dr. Moore: "While some authors condemn mercurializing the patient until a positive diagnosis of syphilis can be made, and claim that nothing is lost by waiting for secondary symptoms, yet I rather incline to the idea that the patient ought to have the benefit of the doubt, and think that something might be gained by mercurializing the patient in primary syphilis, in anticipation of the secondary manifestations, and would not undertake to say that the secondary symptoms might not be aborted altogether if we could feel certain of our diagnosis and press the treatment systematically for a sufficiently long time. The importance in early diagnosis and caution in too sanguine assurance to the patient, while it may not be worth a very great deal to the patient, possibly might be worth a good deal to the doctor. It is not every time that a patient can appreciate the fact that a doctor is not infallible; and mistakes of this sort might turn out to be of some consequence to the doctor. Again, should the patient contemplate early matrimonial alliance it would be of considerable importance."

Dr. Williams: "How long do you keep your patients under treatment?"

Dr. Moore: "If I see the case early in the disease, I begin with mercury, just carrying it far enough to redden the gums without salivating; then I use small tonic doses of mercury for a short time if no secondary manifestations appear. If secondary symptoms are present, I continue the treatment eighteen months at least."

Dr. Stephens: "Do you consider tne two diseases identical?"

Dr. Moore: "No, sir; chancre is the local manifestation of a constitutional disease, while chancroid is only a contagious and local ulcer. I begin the mercurial treatment early in all cases, so as to give the patient the benefit of any doubt in diagnosis, not that I believe in the identity of the two diseases."

Dr. Williams: "Is it not possible that your patients received the virus of both diseases at the same exposure?"

Dr. Moore: "I think not. No induration ever appeared in the cases."

Dr. Williams: "I am at present treating two cases of concealed chancre, the induration being felt through the urethral walls, and enlarged lymphatics are present in the groins. Perhaps this may have been the case with your patients."

Dr. Moore: "No, sir; there were no urethral sores."

Dr. Gibson: "I am treating a case of syphilitic mucous patches of the throat. The true character of these sores was not recognized by the family physician. No specific treatment was used, hence there was no improvement. In these cases I use locally a solution of chloride of zinc (40 per cent), or nitrate of silver on copper wire. I like the latter better than the stick caustic. Internally I use iodide of potassium and bichloride of mercury."

Dr. Moore asked what had been the experience of members in the use of the vegetable combinations, so much spoken of of late; such, for instance, as McDade's succus alterans. Can these preparations be relied upon independent of mercury or iodide potassium? If so, they might bring about considerable revolution in the management of syphilis, especially while we are uncertain about mercurializing our patient for fear he may not have syphilis.

No one had used them; the usual syphilitic treatment with iodide potassium and mercury meeting the approval of all.

